# Assessing and presenting summaries of evidence in Cochrane Reviews

**DOI:** 10.1186/2046-4053-2-81

**Published:** 2013-09-23

**Authors:** Miranda W Langendam, Elie A Akl, Philipp Dahm, Paul Glasziou, Gordon Guyatt, Holger J Schünemann

**Affiliations:** 1 The Dutch Cochrane Centre, Academic Medical Center, University of Amsterdam, Amsterdam, The Netherlands; 2 Department of Internal Medicine, American University of Beirut, Riad El Solh St, Beirut, Lebanon; 3 Department of Urology and Prostate Disease Center, University of Florida, College of Medicine, Gainesville, FL, USA; 4 Malcom Randall Veterans Administration Medical Center, Gainesville, FL, USA; 5 Centre for Research in Evidence-Based Practice, Faculty of Health Sciences, Bond University, Gold Coast, QLD, Australia; 6 Departments of Clinical Epidemiology and Biostatistics and Medicine, McMaster University Health Sciences Centre, Hamilton, ON, Canada

## Abstract

Cochrane Reviews are intended to help providers, practitioners and patients make informed decisions about health care. The goal of the Cochrane Applicability and Recommendation Methods Group (ARMG) is to develop approaches, strategies and guidance that facilitate the uptake of information from Cochrane Reviews and their use by a wide audience with specific focus on developers of recommendations and on healthcare decision makers. This paper is part of a series highlighting developments in systematic review methodology in the 20 years since the establishment of The Cochrane Collaboration, and its aim is to present current work and highlight future developments in assessing and presenting summaries of evidence, with special focus on Summary of Findings (SoF) tables and Plain Language Summaries.

A SoF table provides a concise and transparent summary of the key findings of a review in a tabular format. Several studies have shown that SoF tables improve accessibility and understanding of Cochrane Reviews.

The ARMG and GRADE Working Group are working on further development of the SoF tables, for example by evaluating the degree of acceptable flexibility beyond standard presentation of SoF tables, developing SoF tables for diagnostic test accuracy reviews and interactive SoF tables (iSoF).

The plain language summary (PLS) is the other main building block for dissemination of review results to end-users. The PLS aims to summarize the results of a review in such a way that health care consumers can readily understand them. Current efforts include the development of a standardized language to describe statistical results, based on effect size and quality of supporting evidence.

Producing high quality PLS and SoF tables and making them compatible and linked would make it easier to produce dissemination products targeting different audiences (for example, providers, health policy makers, guideline developers).

Current issues of debate include optimal presentation formats of SoF tables, the training required to produce SoF tables, and the extent to which the authors of Cochrane Reviews should provide explicit guidance to target audiences of patients, clinicians and policy-makers.

## Background

Cochrane Reviews are intended to help providers, practitioners and patients make informed decisions about health care. After collecting, appraising and analyzing the evidence, assessment and presentation of summaries of the evidence support the process of going from evidence to recommendations. The Applicability and Recommendation Methods Group (ARMG) has been largely responsible for developing the methodology for this part of Cochrane Reviews, which deal with interpretation of the results of the reviews and facilitating the uptake of the information they contain. In this paper we will present the different summary presentations, and discuss their rationale, current format, added value and future development. We will focus on Summary of Findings (SoF) tables and plain language summaries (PLS) as they are the main building blocks for dissemination of review results to end-users. We start by outlining the work of the ARMG and the link with GRADE Working Group.

### The applicability and recommendation methods group

#### History

Following an exploratory meeting in 1995 at the Cochrane Colloquium in Oslo, the ARMG was established to look at the applicability of review results to different groups of patients and how to best present the results of Cochrane Reviews so that they would be readily understood and widely used. Key issues included considerations on how to best present a summary of the magnitude of effect for beneficial and harmful effects, transparently identifying predictable causes of heterogeneity in the absolute effects, the influence of individual risk or severity of illness on net effect, and patient’s values and circumstances [1].

The new group, convened by Paul Glasziou and Gordon Guyatt, initially focused on the review and development of methods for addressing these issues, partly supported by a review of past work commissioned by the Australian National Health and Medical Research Council [2]. The group had some input to a section of the Cochrane Handbook, and developed a five-step process that began with a summary of the main benefits and harms.

As The Cochrane Library became more complete and mature, the issues of applicability and recommendations became more important to users of reviews. At the same time, the awareness arose that users need optimal succinct structured and reasonably uniform summaries to understand the key findings of the often comprehensive Cochrane Reviews and to facilitate judgments on applicability. Therefore, in 2004, at the Cochrane Colloquium in Ottawa, Paul Glasziou and Andy Oxman led empirical work at an ARMG workshop exploring contents of a SoF table which would summarize the main beneficial and harmful effects in a tabular format. This idea and a draft SoF table was warmly received.

Encouraged by the work of the ARMG while at the Ottawa workshop, and after being offered co-leadership of the group, Holger Schünemann replaced Paul Glasziou as co-convenor of the ARMG. As there was much overlap among the work of the GRADE working group, more intense work on common aims resulted. For example, Holger Schünemann and Andy Oxman had led the GRADE Working Group in producing the GRADE profiler software (GRADEpro) to produce the SoF tables and integrate with RevMan. Through work under the umbrella of the ARMG and the GRADE working group, the SoF table has been further developed. Since then, there has been a slow but steady uptake of the SoF tables, which have improved the usability of reviews, and implemented a substantial part of the original agenda of the ARMG.

#### Current aim

In 2013, the mission of the ARMG was refined, becoming “to develop approaches, strategies, and guidance that support the dissemination of Cochrane Reviews and their use by a wide range of audience with specific focus on developers of recommendations (including guidance, guidelines, policies) and on healthcare decision makers (e.g. clinicians, policy makers)”.

Specifically, the ARMG provides guidance to optimize the usefulness of Cochrane Reviews by articulating reasons for grading the quality of the evidence and factors to consider when moving from evidence to recommendations. The group also provides detailed guidance to authors of Cochrane Reviews on how to apply the factors, and to users of Cochrane Reviews on how to interpret the judgments made by review authors. This includes guidance on providing the systematic review audience with the information necessary to make judgments about applicability and on making direct statements about applicability. For example, the *Cochrane Handbook for Systematic Reviews of Interventions* explains what the review author can do to help the user to apply the study findings to the population at large or a specific person: “Cochrane review authors must be extremely clear on the population, intervention, and outcomes that they are intending to address. A crucial step is the specification of all patient-important outcomes relevant to the intervention strategies under comparison” [3].

The ARMG also takes responsibility for training the editorial teams of Cochrane Review Groups and review authors to enable the development of SoF tables. Members of the ARMG conduct research on the applicability and the presentation of evidence. In addition, the group disseminates relevant research conducted by its members and other investigators.

Membership, scope of work and objectives of the ARMG overlap with those of the GRADE Working Group (in fact, at the Melbourne Colloquium in 2005, the GRADE Working Group considered whether or not they should merge with the ARMG and operate under the Cochrane umbrella only). The GRADE Working Group began in the year 2000 as an informal collaboration of people with an interest in addressing the shortcomings of grading systems in health care. The working group has developed a common, sensible and transparent approach to grading quality of evidence and strength of recommendations and is working to improve this approach. Many international organizations have provided input into the development of the approach and have started using it (http://www.gradeworkinggroup.org). Through numerous workshops and training events the GRADE working group has both disseminated the SoF tables and enhanced the profile of the ARMG.

## Summary presentation of evidence in Cochrane Reviews

The Cochrane Collaboration launched several initiatives in recent years to develop and evaluate summaries of Cochrane Reviews for different target groups [4]. The SoF table was developed mainly for health professionals [4,5], while different formats of the PLS have been developed mainly for consumers [6,7].

There has been a realization over the last few years that health professionals may not be at much more ease than consumers in understanding and interpreting statistical information, even when presented in a SoF table. At the same time, some consumers might be interested in more detailed information than that presented in a PLS.

### Summary of findings tables

SoF tables are becoming an integral part of Cochrane Reviews by providing a concise and transparent summary of the key findings of a review. At present, SoF tables are more frequently used in Cochrane Reviews than in other systematic reviews and studies have demonstrated that they improve the accessibility and understanding of Cochrane Reviews [4,5]. However, they have been featured in prominent journals, such as *The New England Journal of Medicine*[8].

SoF tables aim to provide a succinct, easily interpretable presentation of the evidence for healthcare providers to make well-informed decisions [9,10]. Critical elements include the confidence in the effect estimates (quality of evidence) and magnitude of effects. The system of assessing the quality of the evidence was developed by the GRADE Working Group [11] and adopted by the Cochrane Collaboration.

#### Format

**Figure 1:**
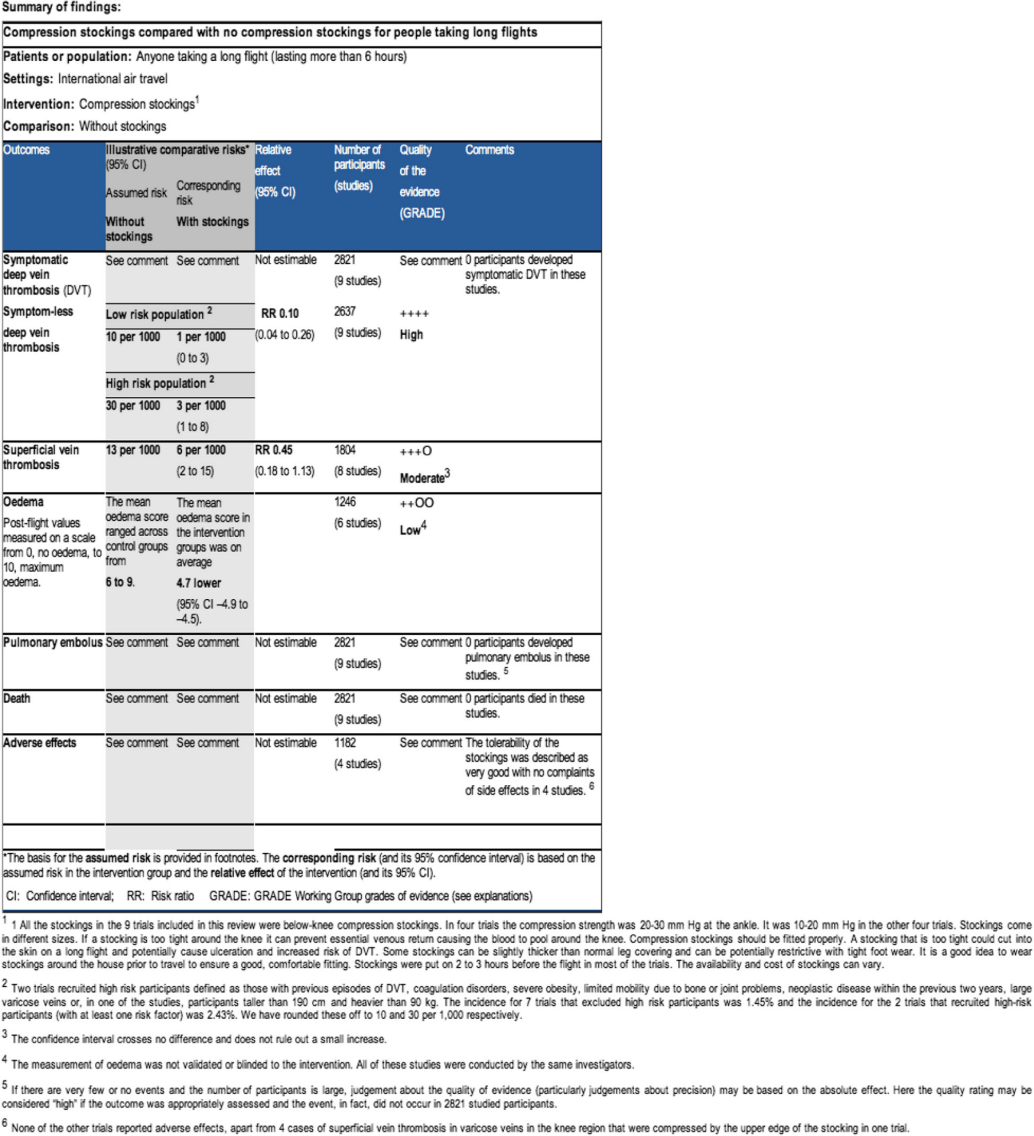
Snapshot of a Summary of Findings (SoF) table.

The current format of the SoF table is the product of several initiatives of the ARMG and the GRADE Working Group to develop and evaluate summaries of Cochrane Reviews for different target groups [12]. Examples of the current format of a SoF table are presented in Figure 1. The types of information that could be included in a SoF table are as follows:

1) A list of all important outcomes; both desirable and undesirable.

2) A measure of the typical burden of these outcomes (for example, control group risk, estimated risk).

3) A measure of the risk in the intervention group, (or alternatively, or in addition, a measure of the difference between the risks with and without the intervention).

4) The relative magnitude of effect.

5) The numbers of participants and studies addressing these outcomes.

6) A rating of the overall confidence in effect estimates for each outcome (which may vary by outcome).

7) And possibly, a comments section that provides clarification and/or additional information.

Supported by the Cochrane Methods Innovation Fund (MIF), the groups are currently working on alternative formats for the SoF tables.

The SoF table differs from GRADE evidence profiles (EP) in the presentation of the quality assessment of each factor that determines the quality of evidence for each outcome (for example, the reason for downgrading due to study limitations). In GRADE EP this is presented in detail, while in the SoF table the quality of the evidence assessment is only provided in key information needed for decision-making. They represent a compromise between simplicity (to make information as easily understandable to a wide audience) and completeness (to make the information and the underlying judgments as transparent as possible, but avoiding unnecessary detail).

#### Outcomes

SoF tables present the outcomes that have been judged to be critical for decision-making. Each of these outcomes should be patient-important rather than a surrogate outcome and their number should be limited to seven, a result of the work done at the workshop at the Cochrane Colloquium in Ottawa in 2004. The choice of seven outcomes, however, is also based on work in the field of psychology, indicating that humans find it difficult differentiating beyond seven factors, and serves the purpose of keeping the presented information manageable [12]. This requires authors of Cochrane Reviews to prioritize outcomes based on their perceived importance (ideally, at a very early stage of the work on their review) as well as potentially aggregating related, yet different, outcomes of approximately equal importance in one outcome measure (for example, combined outcome of gastrointestinal side-effects for patient risk for experiencing either vomiting and/or diarrhea).

Although the SoF table should focus on the patient-important outcomes, there are situations in which there may not be direct evidence for these outcomes. In that case, reviewers should present their inferences regarding treatment effect from the surrogate outcomes measure; and this should be clearly labeled. The authors would do this by using the best estimate of the baseline risk for the patient-important outcome (see below) and then applying the relative effect from the surrogate outcome.

Another special situation may arise when the quality of evidence is low or very low for the relevant, patient-important outcome and a related, indirect outcome measure exists that is (rightly or wrongly) perceived to be highly relevant by the clinical community. In such situations, indirect evidence may be presented, but authors should be explicit about its role as a surrogate measure.

In general, SoF tables should present the highest quality of evidence available for a given outcome. When the quality of two bodies of evidence is similar (for example, randomized trials and observational studies), SoF tables may include summaries of both. It is also possible that review authors found no published evidence regarding one or more critical or important outcomes.

#### Baseline risk

An important issue for decision-making is the determination of a baseline risk for a particular patient-important outcome. GRADE recognizes that the patients in randomized trials may be unrepresentative of the general population (for example, they may have been selected for being at high risk, thereby reducing sample size requirements) and that this baseline risk varies for prognostic subgroups. Therefore, calculating absolute measures of effect size directly from the data from randomized trials may be misleading. Instead, baseline or control group risk should be drawn from well-designed observational studies if available [12].

If high quality observational studies are not available, GRADE suggests using the median risk (rather than the weighted average, which is influenced by outliers) among the control groups in the included studies. If there is important variation on control group risk, GRADEPro software (computer program developed by Jan Brozek, Andy Oxman and Holger Schunemann) (provides the opportunity for authors to present a range of risks upon which the calculation of absolute effect size measures is then based. To date, GRADE has shied away from also seeking to quantify the uncertainty surrounding the estimates of baseline risk [12]. This has been a pragmatic decision to avoid additional complexity, which may make the systematic review process unmanageable.

#### Effect size presentation

Based on studies of consumer numeracy, effect size presentation as natural frequencies, that is, as event per 100 patients, for example, is preferred over presentation of relative risks [13]. Recent data (from ongoing research), however, suggest percentages may do as well and sometimes better. User testing of evidence profiles of guideline panelists randomized to four different presentation features has further documented the value of presenting absolute risk differences [14]. Whether or not to include them was an issue of intense debate at the GRADE working group meeting, held in conjunction with the Cochrane Colloquium in Sao Paolo (2007). Absolute measures of effect should be presented in conjunction with confidence intervals reflecting the underlying precision and indicate the length of follow-up to which estimates refer.

Whereas relative and absolute effect sizes used to report the results of dichotomous measures are very familiar to clinical audiences, summary measures for continuous outcomes present particular challenges to interpretation [15]. The most common approach divides the difference in means in each study by its standard deviation and presents pooled results in standard deviation units (standardized mean difference). Its drawbacks include the vulnerability to heterogeneity and difficulties in interpretation.

One approach advocated in the interest of improved interpretability is the reporting in minimal important difference (MID) units, when the MID is known. Another related approach is to use the MID as the threshold value for converting a continuous outcome into a binary outcome and then presenting relative and absolute effect sizes [15].

#### Uptake of SoF tables

Since 2008, the uptake of SoF tables in Cochrane Reviews has increased steadily. In Issue 1, 2009 (quarterly issue) of *The Cochrane Library*, 3 reviews included a SoF table, and this number had increased to 94 reviews in the Issue 1 to 3, 2012 (monthly issues). In March 2012, a total of 502 Cochrane Reviews included one or more SoF tables. The quality of this set of SoF tables is currently being evaluated by the ARMG. As of September 2013, almost one thousand reviews include a SoF table.

According to the Methodological Standards for the conduct of new Cochrane Intervention Reviews, including a SoF table is highly desirable and assessing the quality of the body of evidence is mandatory for new Cochrane Intervention Reviews.

#### Added value of SoF tables

The added value of SoF tables was first evaluated in 2005, in an unpublished pilot study headed by Gunn Vist in collaboration with Andy Oxman, Paul Glasziou, Julian Higgins and Holger Schünemann. Twenty authors from 17 Cochrane Review Groups (CRGs) were asked to construct a SoF table for their new or updated review. The authors spent on average an additional 4 hours (range 2 to 40 hours) on their review in order to do this. In general, the authors reported that the layout of the SoF Table was clear, and that presenting the review results in a SoF table was found to be helpful. Of the 17 CRGs, 11 concluded that the accessibility of the review was increased, 5 CRGs concluded that the quality of the review was improved and 1 CRG rephrased the conclusions. Most CRGs experienced software difficulties.

The added value of SoF tables was also tested in users of Cochrane Reviews. Two small randomised trials found that including a SoF table in a systematic review improved the user understanding and rapid retrieval of key information [5]. However, the uptake of SoF by review authors has not been optimal. There may be several reasons for this. The standard table does not provide review authors with enough flexibility to accommodate for different kinds of reviews. Some people also perceive the tables to be compact and full of data making them too complex for the users (see Figure 1).

During development, user testing of these tables had revealed one of the main challenges as a “tension between achieving precision and simplicity” [5], where precision refers to comprehensiveness. Comprehensiveness, however, to an untrained eye, can quickly become visual clutter that camouflages the main messages.

The SoF development team addressed this tension by using a layered approach, allowing some parts of the table to emerge as more important to the eye than other parts, through typographical and color differentiation. However, this typographical layered approach was largely not implemented due to the difficulty of technical implementation in website and PDF formats, compromising the balance of precision and simplicity. The full extent, however, to which SoF tables aid, or change, decision making has not yet been formally investigated.

#### Further and future developments

Currently, the ARMG and GRADE Working Group are working on further development of the SoF tables in two research projects. One research project, funded by Cochrane Methods Innovation Fund, evaluates the degree of acceptable flexibility beyond standard presentation of SoF tables. This evaluation includes investigating which alternative columns are acceptable to decision makers and should be included as alternatives in SoF tables, for example, the addition of risk differences and number needed to treat instead of the currently used columns of assumed and corresponding risk. This evaluation will also include development of descriptions for outcomes that could not be pooled, which columns can be collapsed and which comparisons should be described in the primary SoF tables. The second aim of the project is to provide guidance on the standardization of comments and footnotes for SoF tables, with a focus on the explanations for downgrading and upgrading the quality of a body of evidence. The third aim is to develop guidance on what information to include in SoF tables in diagnostic test accuracy reviews. The results of this work, expected at the end of 2013, will be integrated in updated training material to provide optimal guidance to reviewer authors and users of reviews.

**Figure 2:**
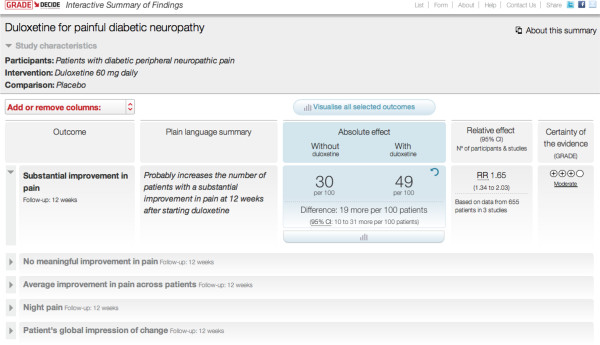
Snapshot of an interactive Summary of Findings (iSoF) table.

The DECIDE project, initiated and developed by the GRADE working group, which runs from 2011 to 2015 (http://www.decide-collaboration.eu), attempts to take advantage of technological advances to improve the SoF table. As a result, the interactive SoF table (iSoF) uses electronic presentation of information to reconcile precision and simplicity through a layered approach to information presentation. The top layer presents basic information, while the deeper layers allow access to more details on demand. In addition, the user has control over how many and which outcomes the table displays and in which format (words, numbers, graphics) (see Figure 2).

The iSoF also has a column to express results in a narrative format in addition to statistical formats, or in lieu of these when statistical data are not available. The multiple representation possibilities make the table more flexible for presenting data from different kinds of reviews.

Additional advantages of the iSoF include an interactive presentation and explanation of confidence intervals and other statistical terms, and a responsive format for printing and for displaying on different sizes and types of electronic devices.

Future plans include the translation of the iSoF into different languages. The iSoF will be incorporated in GRADEpro and possibly other interfaces (for example, Cochrane Reviews, guideline products, electronic medical records). The different versions would present the same core information in potentially variable formats.

### Plain language summary

The plain language summary (PLS, formerly called ‘synopsis’) aims to summarize the results of a review in such a way that consumers of health care without a medical background can readily understand them. The current format of the PLS was informed by a qualitative study among consumers [6]. PLS has two parts: a title and a body of test. In an effort to better disseminate Cochrane evidence synthesis among consumers, efforts are underway to include a PLS in PubMed Health, a freely accessible service by the US National Library of Medicine. Ongoing improvement efforts are being directed towards assuring consistency between the review and the PLS, implementing short yet concise titles and reconsidering the current word count limit of 400 words, while maintaining understandability.

Example of a plain language summary [16]: compression stockings for preventing deep vein thrombosis (DVT) in airline passengers.

In the last few years, there has been increasing interest in whether compression stockings (sometimes called ‘flight socks’) reduce the risk of DVT (blood clots in the legs) and other circulatory problems in airline passengers. The stockings are worn throughout the flight and are similar to those known to be effective in patients lying in bed after an operation. By applying a gentle pressure, to the ankle in particular, compression stockings help blood to flow. Pressure combined with leg movement helps blood in superficial veins to move to the deep veins and back to the heart. The blood is then less likely to clot in the deep veins, which could be fatal if the clot moves to the lungs.

Wearing compression stockings resulted in a very large reduction in symptomless DVT among airline passengers who were allocated to wear compression stockings compared to those allocated not to wear such stockings. People who wore stockings also had much less discomfort and swelling in their legs (edema) than those who did not wear them.

These conclusions were based on nine trials, which studied over 2,800 people, about half of whom were randomly assigned to wearing stockings for a flight lasting at least seven hours while the other half did not. None of the passengers developed a DVT with symptoms (slowly developing leg pain, swelling and increased temperature) and no serious events (a blood clot in their lungs (pulmonary embolus) or dying) were reported. Passengers were carefully assessed after the flight to detect any problems with the circulation of blood in their legs, even if they had not noticed any problems themselves. There was a big difference in symptomless DVT between the two groups, equivalent to a reduction in the risk from a few 10s per thousand passengers to 2 or 3 per thousand. Not all the trials reported on possible problems with wearing stockings but in those that did, the researchers said that the stockings were well tolerated, without any problems.

In connection with the SoF tables, research has been conducted on how to communicate the results of systematic reviews to consumers and how these are perceived and understood [6,7,17]. This work is currently feeding into the Plain Language Expectations for Authors of Cochrane Summaries (PLEACS) project. The PLEACS group has developed a set of minimum criteria for the content of PLS (http://www.consumers.cochrane.org/PLEACS). The purpose is to ensure that authors convey the key question and findings of the review in a succinct and clear manner to consumers. The group is also working on improving the narrative reporting of results by testing the inclusion of headings and numbers.

Current efforts include the development of a standardized language to describe statistical results based on effect size and quality of supporting evidence [6]. This standardized language could be used in PLS and also in abstracts of Cochrane Reviews. The iSoF project is prototyping the use of PLS standardized sentences in their own column in the table, providing the reader with a narrative explanation of the gist of the results, next to the numbers. The Collaboration is currently exploring how standardized language could contribute to ensuring the quality of translation of PLS and abstracts.

### Future direction of PLS and SoF tables

A major future direction for the presentation formats of PLS and SoF tables is making both of them usable by both audiences and cross-linking them for those who are interested in both. One could conceptualize PLS and SoF tables, in particular, as the basic building blocks for dissemination to end-users. Producing high quality PLS and SoF tables and making them compatible and linked would make it easy to produce dissemination products targeting different audiences (for example, providers, health policy makers [17], the press, guideline developers).

Furthermore, to avoid the risk of a one-size-fits-all approach to all recommendations, issues of who to apply results to and attempting to divide them into different risk groups (low, medium, high) deserves more work in the future.

### Training, support and tools

Among the core functions of the ARMG are training, providing specialist advice and contributing to software development. Following the first GRADE Working Group articles published in 2003 and 2004, the *British Medical Journal* published a series of papers on the GRADE approach in 2004, authored by the GRADE Working Group. Subsequently, in 2011, publication of a series of 20 papers in the *Journal of Clinical Epidemiology* started. The first series provides the basics of the GRADE approach, while the second series offers detailed guidance on applying GRADE and constructing SoF tables. Among the (local) training activities are the GRADE/SoF workshops run by the Dutch and German Cochrane centers and McMaster University, and pre-conference and conference workshops at Cochrane Colloquia and the meetings of the Guidelines International Network. Examples of online training initiatives are the McMaster GRADE Online Learning Modules (cebgrade.mcmaster.ca), YouTube videos and the slidecast presentations of Cochrane Training. In February 2013, the ARMG launched a question and answer webinar series. To increase the capacity of training and support, the ARMG is currently creating a network of members who can be consulted for advice and support on making SoF tables and GRADE profiles. Most of the support is currently provided by members of the ARMG at McMaster University who have already helped many authors of Cochrane Reviews. The new version of GRADEpro, which will include training exercises and links to training material, will be part of the Guideline Development Tool (http://www.guidelinedevelopment.org), a comprehensive new tool for developing evidence-based guidance. An update of the currently available two chapters of the ARMG in the Cochrane Handbook for Systematic Reviews on Interventions is expected in 2013 [3,18].

### Current debates and challenges

Current issues of debate and controversy include optimal presentation formats of SoF tables, the training and perceived additional effort involved in using GRADE, and the extent to which authors of Cochrane Reviews should provide explicit guidance to target audiences of patients, clinicians and policy-makers. The first of these should be settled by ongoing empirical investigation and the interactive SoF table. The other two issues may be more challenging to solve. Criticisms of GRADE include its complexity, the time involved in its application, and the extent to which its application yields ratings of low confidence in effect estimates. GRADE proponents argue that the complexity is not in GRADE but in the issues that GRADE has brought to light (for example, complex judgments related to precision, directness and consistency, and thresholds for when to rate down confidence regarding these components and for risk of bias). If one is going to address, rather than ignore, these issues, GRADE provides a structure that simplifies, rather than increasing the complexity. GRADE proponents also argue that the time required is in preparing summaries that allow one to address the issues of evidence evaluation, rather than in the evaluation itself. GRADE may indeed lower the workload by providing a structure to what has often been an unstructured and, therefore, more disorganized and laborious effort. With respect to the issue of the likelihood that application of GRADE will result in conclusions of low confidence in effect estimates, paucity of high quality evidence is not the fault of GRADE or the SoF table.

More importantly, GRADE and ARMG are attentive and respectful to these concerns. Where this attentiveness plays out vividly at the moment is in the cautious approach to dealing with uncertainty related to baseline risk or diagnostic test accuracy studies. Greater attention to this up to now relatively neglected area will increase complexity, may increase time required, and will increase the likelihood of ratings of low confidence in effect estimates. GRADE is currently working on advancing our conceptual understanding of these issues and providing a way to advance their consideration while minimizing associated burdens on systematic review authors and guideline developers.

The Cochrane Collaboration is explicit that systematic review authors should not make recommendations. At the same time, audiences seek guidance on how results should be applied, and Cochrane provides the opportunity for authors to reflect on the implications of their review for clinical practice and public policy. These competing considerations create tension. A potential solution in the clinical, and perhaps the health policy area, is for authors to highlight particular constellations of values and preferences and their implications, given the results, for particular courses of action.

## Abbreviations

ARMG: Applicability and Recommendation Methods Group; CRGs: Cochrane Review Groups; DVT: Deep vein thrombosis; EP: Evidence profiles; GRADE: Grading of recommendations assessment, development and evaluation; iSof: Interactive summary of findings table; MID: Minimal important difference; MIF: Methods innovation fund; PLEACS: Plain Language Expectations for Authors of Cochrane Summaries; PLS: Plain language summary; SoF: Summary of Findings.

## Competing interests

All authors are members of the ARMG and GRADE Working Group. GG and HS are co-convenors of the ARMG and co-chairs of the GRADE Working Group.

## Authors’ contributions

ML, EA and HS developed the outline of the manuscript. All authors contributed to the writing of the manuscript and critically reviewed the manuscript. All authors read and approved the final manuscript.
